# Genetic conflict and the origin of multigene families: implications for sex chromosome evolution

**DOI:** 10.1098/rspb.2023.1823

**Published:** 2023-11-01

**Authors:** Emiliano Martí, Amanda M. Larracuente

**Affiliations:** Department of Biology, University of Rochester, Rochester, NY 14627, USA

**Keywords:** intragenomic conflict, ampliconic genes, meiotic drive, sex chromosomes, Y chromosome, genome evolution

## Abstract

Sex chromosomes are havens for intragenomic conflicts. The absence of recombination between sex chromosomes creates the opportunity for the evolution of segregation distorters: selfish genetic elements that hijack different aspects of an individual's reproduction to increase their own transmission. Biased (non-Mendelian) segregation, however, often occurs at a detriment to their host's fitness, and therefore can trigger evolutionary arms races that can have major consequences for genome structure and regulation, gametogenesis, reproductive strategies and even speciation. Here, we review an emerging feature from comparative genomic and sex chromosome evolution studies suggesting that meiotic drive is pervasive: the recurrent evolution of paralogous sex-linked gene families. Sex chromosomes of several species independently acquire and co-amplify rapidly evolving gene families with spermatogenesis-related functions, consistent with a history of intragenomic conflict over transmission. We discuss Y chromosome features that might contribute to the *tempo* and *mode* of evolution of X/Y co-amplified gene families, as well as their implications for the evolution of complexity in the genome. Finally, we propose a framework that explores the conditions that might allow for recurrent bouts of fixation of drivers and suppressors, in a dosage-sensitive fashion, and therefore the co-amplification of multigene families on sex chromosomes.

## 1. Introduction

Organisms display remarkable adaptations that ensure viability and reproduction. These are products of natural selection operating at the organismal level, which requires cooperation among the thousands of genes that build an individual. This is straightforward in asexual lineages, where all genes inexorably share the same evolutionary fate with their host. On the other hand, adaptation in sexually reproducing organisms relies on the fairness of meiosis [1]—a marvellous device that ensures both reduction of ploidy and fair Mendelian segregation of chromosomes during gametogenesis. An equal probability of segregation between the parental alleles maximizes the efficacy of natural selection [2]. As a result, ignoring mutation and genetic drift, only alleles that confer some benefit to their host should be overrepresented in future generations. However, sex also creates the opportunity for intragenomic conflict [3]. Sexually reproducing populations are susceptible to the invasion of segregation distorters, selfish genetic elements (SGEs) that subvert the mechanism of meiosis to increase their own transmission, even when this poses negative consequences to the organism [1,4–7].

Male segregation distorters are systems with (at least) two main components—a driver allele at a *trans*-acting locus that undermines the transmission of a sensitive allele at a *cis*-acting target. Linkage disequilibrium (LD) between the driver and target loci is essential for the spread of the driver in the population [8,9]. Otherwise, ‘suicidal’ combinations that distort against themselves are created by recombination [10]. Therefore, autosomal drivers are often found in regions of low recombination (e.g. close to pericentric heterochromatin) and/or can acquire chromosomal inversions that suppress recombination [11–14]. Sex chromosomes, however, have properties that make them inherently susceptible to intragenomic conflicts [15–17], including invasion by multilocus drive systems [18,19]. For one, sex chromosomes usually do not recombine over much of their length, reducing the risk of suicidal combinations [20]. For another, most X- and Y-linked loci in species with differentiated sex chromosomes lack homology throughout most of their length, providing more opportunities for the driver to exploit *cis*-acting target loci. Together, these characteristics predict that meiotic drivers will be far more common in sex chromosomes compared to autosomes [18].

The invasion of segregation distorters can have negative consequences for the fitness of individuals and populations [15,21,22]. First, a driver allele on one chromosome impairs the function of gametes carrying its homologous counterpart, and therefore imposes a fertility cost (reviewed in Zanders & Unckless [23]). Second, deleterious mutations linked to the driver can hitchhike to higher frequencies in the population [24,25]. Third, sex-linked distorters skew population sex-ratios. This effect elicits selection to re-establish a close to 1 : 1 Fisherian sex-ratio—as one of the sexes becomes rarer, individuals of that sex would have a higher reproductive value than those of the more abundant sex [26]. If not kept in check, sex-ratio distorters can cause population extinction [15,21]. Therefore, SGEs, like segregation distorters, and their host genomes have conflicting evolutionary interests. In response, host genomes can evolve unlinked suppressors that neutralize their effects [15,27–30]. In addition, there is selective pressure for drivers to recruit genetically linked enhancers that increase the strength of distortion [14,31]. These conflicts trigger coevolutionary arms races, in a host–parasite fashion, with bouts of innovation and counter-innovation that have far-reaching evolutionary consequences for genome regulation, sequence and organization, gametogenesis, recombination, reproductive strategies and even the origin of intrinsic postzygotic hybrid incompatibilities [7,19,20,32–34].

One particular outcome of dosage-mediated intragenomic conflicts—that is, when the phenotype of segregation distortion correlates with the number of copies or level of expression of the driver—on genome organization is the origin of redundancy [35]. Recurrent cycles of drive and countervailing selection can lead to the evolution of repeated structures. Here, we focus on an emerging aspect of conflicts between sex chromosomes: the acquisition and massive co-amplification of multigene families. Several properties of these systems suggest a conflict over transmission [36,37]. While direct evidence for conflict between co-amplified gene families is limited to a few systems [35,38,39], the increasing availability of high-quality genome assemblies, particularly of the sex-limited chromosomes, suggests that conflicts involving co-amplification of gene families between sex chromosomes might be a widespread phenomenon. Recent advances in the ‘genomics era’ highlight the importance of sex chromosome biased mutational spectrum fuelling intragenomic conflicts, as well as consequences for genome organization and genetic diversity.

## 2. Conflicts mediate co-amplification of multigene families

Gene loss is a hallmark of Y chromosome evolution [40–42]. However, comparative analyses across multiple taxa indicate that the acquisition of genes by Y chromosomes from other genomic locations is also a rather common phenomenon [43–47]. The traffic of genes to the Y chromosome can resolve antagonistic conflicts over traits that increase male fitness but could be detrimental for females, as is the case for male fertility and spermatogenesis-related genes [3,48–51]. Some of these newly acquired genes subsequently amplify in copy number to produce ‘ampliconic’ gene families [52–57]. The amplification could be favoured to circumvent the highly repetitive and/or inert heterochromatic environment imposed by the Y chromosome. For one, extra gene copies, including fragmented ones, could serve as a template for non-allelic exchange (i.e. gene conversion, unequal recombination) and therefore prevent pseudogenization [53]. Amplification might also be favoured because the Y chromosome is epigenetically repressed [58]—gene family expansion could be selected for increasing gene products in a dosage-sensitive manner [37,59]. Alternatively, gene amplification could also be the product of non-deterministic processes—the highly repeated content of the Y chromosome is prone to non-homologous exchange events that can cause expansion or deletions [60,61]. In addition, these duplications could be neutral or even slightly deleterious. The reduced effective size and the absence of recombination on the Y chromosome renders natural selection inefficient, even in very large populations [26,42,62–64]. Thus, slightly deleterious mutations in the Y (or W) chromosome can be effectively neutral [40].

Y-linked ampliconic genes might then have male-biased expression and fertility-related functions or are simply amplified and maintained by stochastic processes. In addition, the increasing availability of high-quality genome assemblies is revealing a different category—some Y-linked ampliconic genes also have co-amplified paralogs on the X chromosome [52,55,61,65–67]. X/Y co-amplified gene families share many characteristics with other Y-linked ampliconic sequences. For instance, sex-related genes often evolve rapidly, and these ampliconic genes are no exception [61,68,69]. However, rather than sex-specific fitness functions, other features of X/Y co-amplified gene families are consistent with a history of intragenomic conflict.

### (a) Neo-sex chromosome are havens for intragenomic conflicts

Sex chromosomes inherently provide the opportunity for conflicts over transmission—LD is normally complete across most of their length, preventing the generation of recombinant ‘suicidal’ chromosomes [10,18,20]. However, the onset of the intragenomic conflict is also contingent on the mutation rate to new drivers. In species with differentiated sex chromosomes, this depends on the number and nature of genes present on the X and Y chromosomes, or the rate at which potentially exploitable autosomal genes become sex-linked by duplication. Neo-sex chromosomes—in which a whole, or segment, of an autosome is translocated to one or both preexisting sex chromosomes—in turn, provide a rich substrate for the emergence of genetic conflicts. Following the chromosomal fusion and cessation of recombination, an entire autosomal gene set immediately becomes sex-linked and thus has sex-restricted or -biased transmission, and the potential to acquire selfish mutations.

Employing male to female coverage ratios, Ellison & Bachtrog [65] recently discovered X/Y co-amplified gene families in several species of *Drosophila*: 11 out of 26 species have the ancestral *Drosophila* sex chromosome (Muller element A), whereas 15 have independently formed neo-sex chromosome systems (products of fusions of *different* autosomes with one or both sex chromosomes). After becoming sex-linked, the chromosome arm starts to evolve the properties of ancestral sex chromosomes, including gene loss, accumulation of repeats and recruitment of modifiers of recombination [40,42,70]. The neo-sex chromosomes in this sample originated at different times and therefore reflect different stages of this process. Strikingly, nine of the ten species with X/Y co-amplified gene families also harbour neo-sex chromosomes, and the parental gene was present on the autosome that formed the neo-sex chromosomes [65]. In these species, 34 different genes (out of 35, if we consider the ampliconic gene family in the species with ancestral sex chromosomes) were co-amplified between sex chromosomes. This study may represent only the tip of the iceberg, due to the limitations of short-read sequencing data and reliance on the *Drosophila melanogaster* reference genome for annotations [65]. High-quality genome assemblies offer a more unbiased gene discovery, as shown in the *Drosophila miranda* neo-Y, in which the total number of genes has doubled since it evolved [55,71]. Since its formation approximately 1.5 Ma, the *D. miranda* neo-Y has gained over 3200 protein coding genes, in addition to the approximately 3000 present in the ancestral autosome (Muller element D) [55]. Of the Y/neo-Y total ampliconic genes, 2036 were co-amplified—that is, their X-linked paralogs were also amplified—and derived from 94 different ancestrally single-copy protein coding genes [55].

### (b) X/Y co-amplified gene families with gonad-enriched functions suggest underlying conflicts

Many co-amplified genes are testis-expressed and have predicted functions in meiosis and spermatogenesis [37,52,65,72,73]. Their parental genes are also enriched for expression in gonads in related species, further suggesting that they have roles in gametogenesis [65]. Even more remarkable is the *independent* co-amplification of the same genes in different lineages [65]. In members of the *Drosophila obscura* group, the autosomal genes *S-Lap1* and *GAPsec* were amplified independently in both sex chromosomes after becoming sex-linked due to the formation of neo-sex chromosomes. *GAPsec* is a GTPase activating protein, which is reminiscent of *Sd-RanGAP*, the main driver in the well-known autosomal male drive system of *D. melanogaster* called *Segregation Distorter* ([74], reviewed in [75]). *RanGAP* has roles throughout the cell cycle but is best studied for its role in nuclear transport, where its normally cytoplasmic localization is important. The driver, *Sd-RanGAP*, is a partial duplication of the parent gene *RanGAP* [74]. The mislocalization of *Sd-RanGAP*, and its presumed effect on nuclear transport, may be the basis of the segregation distortion phenotype [76]. The nuclear transport pathway in *Drosophila* seems to be intrinsically susceptible to selfish mutations—several components often show whopping signatures of rapid nucleotide evolution, and in some cases are involved in hybrid incompatibilities [77,78].

### (c) Rampant amplification of Y-linked paralogs

**Figure 1.**
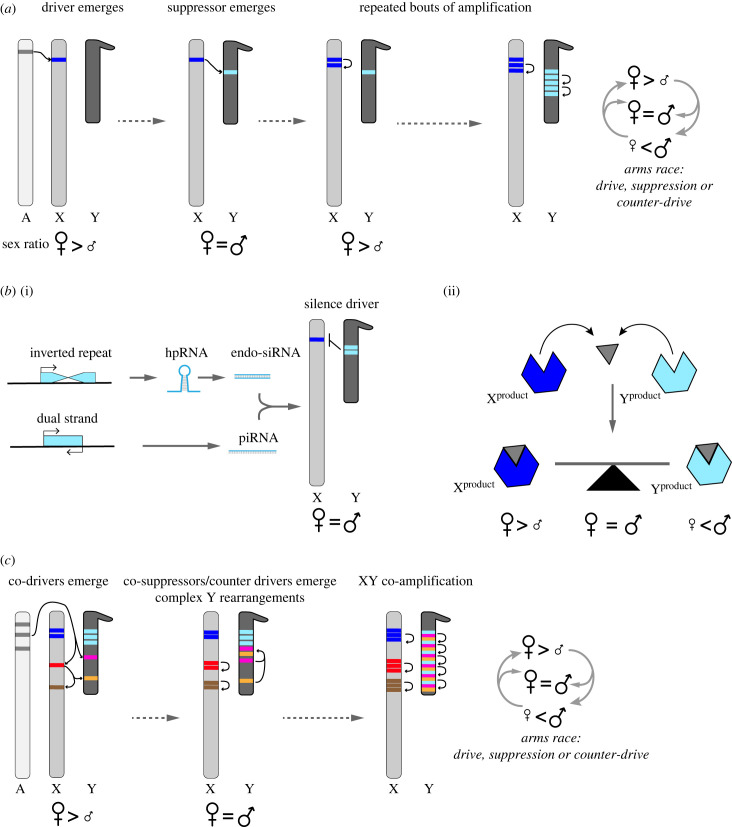
Dosage-mediated conflicts can promote the evolution X/Y co-amplified gene families. (*a*) An autosomal gene (A) duplicates to the X chromosome. The new X-linked duplication evolves into a meiotic driver and causes sex-ratio distortion in favour of females. The fitness consequences of drive for individuals and populations will elicit selective pressure for the evolution of a suppressing or counteracting Y-linked mutation that emerges through a duplication event onto the Y chromosome, restoring equal sex-ratios. The X-linked driver can escape suppression through gene duplication to amplify copy number, skewing sex-ratio towards females once again. Selection would then trigger an evolutionary arms race leading to the formation of co-amplified gene families through recurrent cycles of drive and suppression, or counter-drive, in the case where the Y-linked paralog amplification can skew sex-ratio towards males in a dosage-sensitive manner. The amplification of Y-linked paralogs tends to be greater, which may be a result of its unique mutational spectrum (see §3b). (*b*) Suppression can emerge from: (i) RNA interference mechanisms where a Y-linked duplicate acquires structural rearrangements that give rise to a Y-linked inverted repeat capable of producing an hpRNA/endo-siRNA, or dual-stranded transcripts that give rise to piRNAs, that silence the driver or (ii) interaction of products (e.g. proteins, RNA) from both the X- and Y-derived genes in a dosage-sensitive manner. In this case, the driving sex chromosome encodes a product that causes sex-ratio distortion, and its counterpart encodes a negative regulator of drive shifting sex-ratios in either direction depending on the relative abundance of X- and Y-linked products. (*c*) Over time, conditions ripe for XY co-amplification can lead to the evolution of genetic complexity. In a genetic background like the one illustrated in (*a*), the X and Y chromosomes can acquire novel X-drive loci and Y-linked suppressors (or counter drivers) resulting in an increasingly complex gene network. Unique mutational properties on the Y chromosome might facilitate the incorporation of different gene families into higher-order-repeat structures that can be amplified through unequal exchange, increasing the complexity of the system. The X-linked paralogs of these gene families remain organized into discrete clusters. While here we illustrate drive emerging from autosome-to-sex chromosome duplications, for neo-sex chromosomes, there are many more potential drivers and suppressors.

When genes are co-amplified on both the X and Y chromosomes, the Y-linked paralogs are typically amplified to higher copy number (e.g. [53,55,61,65,66,79]; figure 1*a*). Some of the most dramatic examples of rampant Y-linked paralog amplification are in cases with known genetic conflicts like *Sly* in mice [52] and *Su(Ste)* in *D. melanogaster* [35,80–82]. This biased amplification of Y-linked paralogs may be a result of the high repeat density of the Y chromosome. Repetitive DNA sequences can experience rapid copy number change due to non-homologous exchange [83] between repeated sequences like tandem repeats, transposable elements (reviewed in [70]), or duplicated genes (reviewed in [84]). Unequal exchange between sister chromatids at duplicated genes can lead to dramatic amplification in gene copy number over short periods of time [83]. Gene conversion can lead to the homogenization of gene copies and might protect them from accumulating deleterious mutations (e.g. [49,53,85–87]). For example, 30% of the human male-specific part of the Y chromosome is composed of ampliconic genes with a palindromic organization [88]. Due to their organization, some of these ampliconic gene families show 99.9% sequence similarity due to gene conversion [88].

### (d) Recruitment of small RNA pathways

One recurring theme emerging from studies of intragenomic conflicts is the involvement of RNA interference pathways [89], where small RNA-producing loci can be involved in antagonistic interactions (reviewed in [90]; figure 1*b*). In the case of male meiotic drive, paralogs of the driver can acquire the capacity to suppress drive through production of endogenous small interfering RNAs (endo-siRNAs) (e.g. the Winters sex-ratio system of *Drosophila simulans*) or Piwi-associated RNAs (piRNAs; the *Su(Ste)* repeat in *D. melanogaster*).

The RNAi machinery (e.g. *Dicer-2* and *Argonaute 2*) processes double-stranded RNA (dsRNA) templates into 21-nt endo-siRNAs that can silence their targets through base complementarity (reviewed in [91]). The sources of dsRNAs can be from inverted repeat structures, bidirectional transcription or antisense transcripts from unlinked genomic loci. Recent studies reveal networks of evolutionarily young hairpin RNAs that have predicted X-linked targets in *Drosophila* species [92]. These hpRNAs may be involved in intragenomic conflicts over sex-ratio distortion. A network of novel hpRNAs in *D. simulans* gives rise to endo-siRNAs that are suppressors of the Winters (*Nmy*; [93]) and Durham (*Tmy*; [94]) sex-ratio drive systems. The driver loci in these systems have emerged, amplified and diversified in the *simulans* clade (*D. simulans*, *D. mauritiana* and *D. sechellia*) but are absent from their closely related species, *D. melanogaster* [95,96].

Similarly, the piRNA pathway has a role in mediating suppression of sex-linked gene families in *D. melanogaster* [97]. The piRNA pathway involves germline-restricted 22–30-nt single-stranded RNAs best studied for their roles in silencing transposable elements (e.g. [98]). But it also targets other repeated sequences [97], including gene families involved in conflicts. *Stellate* (*Ste*) is an X-linked tandemly repeated gene that encodes a protein homologous to the beta subunit of casein kinase II [99,100]. When expressed, it leads to the accumulation of nuclear and cytoplasmic protein that aggregates into crystalline structures, causing male sterility [100]. Some studies indicate that *Ste* expression also causes female-biased sex-ratio in subfertile males [35,101]. However, *Ste* expression is suppressed by a paralog, *Suppressor of Stellate* (*Su(Ste)*), that is independently amplified in the Y chromosome [81,82]. The Y-linked copies acquired an insertion of a 1360 DNA transposon [102] that induces the production of antisense transcripts and subsequent processing of *Su(Ste)* by the piRNA pathway [97,103].

While RNAi pathways seem to be an emerging theme in conflicts and conflict resolution, species may differ in how they mediate these conflicts. Mutations in the RNAi pathway genes *Dicer-2* and *Argonaute 2* are male-sterile in *D. simulans*, but not in *D. melanogaster* [94]. By contrast, the piRNA pathway seems to be more important in *D. melanogaster*. In this species, the Y-linked *petrel* locus corresponds to a piRNA cluster that silences the X-linked gene *pirate,* a SUMO (small ubiquitin-related modifier) protease. In *Drosophila mauritiana*, *pirate* is also targeted for silencing, but the Y-linked locus generates endo-siRNAs instead [104]. While the nature of the conflict remains to be determined, these results implicate convergent evolution for the suppression of *pirate*, and highlights that there may be species-specific strategies to mediate intragenomic conflicts.

Some primary components of piRNA and endo-siRNA pathways, including downstream genes with chromatin functions, are involved in duplication events to sex chromosomes [55]. It is thus possible that components of RNAi pathways themselves, in addition to their small RNA products, are caught up in genetic conflicts.

## 3. Consequences: evolutionary dynamics and rapid structural evolution

The resolution of intragenomic conflicts has consequences for sex chromosome organization and the functional specialization of sex-linked genes. For instance, the recruitment of small RNA pathways can affect sex chromosome organization, as rearrangements can be involved promoting antisense transcription of one set of paralogs, and therefore, suppression of its counterpart. This seems to be the case for the X-linked *Ste* and Y-linked *Su(Ste)* co-amplified loci, which are considered to be a relic meiotic drive system in *D. melanogaster* [35,80]. The stepwise evolution of *Su(Ste)*—through acquisition of testis-specific antisense transcripts homologous to *Ste*—led to the evolution of two large repetitive arrays on the Y chromosome. Remnants of this same gene family’s duplicates, including independently amplified genes and pseudogenes, perhaps each with their own history of conflict, exist on the sex chromosomes of closely related species in the *D. simulans* clade [61].

### (a) Conflict resolution can affect genetic complexity

Aside from the rapid structural change on sex chromosomes, conflict resolution may lead to the evolution of genetic complexity to the extent where drive systems, and thus genomes, become *overwired* (to borrow a term from Frank [105]). Bouts of selfish substitutions and gene expansion, followed by countervailing selection and conflict resolution, progress like a ratchet. As long as the drive-suppressor system is not dead—that is, that has not yet accumulated deactivating mutations—organismal fitness hinges on the maintenance of some of the components of the system. For instance, derepression of *Ste* renders males sterile [106]; as a consequence, the maintenance of fertility now depends on the perpetual production of piRNAs from *Su(Ste)* in the male germline. In addition, the redundant nature and organization of these multigene families make them prone to expansion and contraction by non-homologous recombination. Once fixed, it may take more evolutionary time for dosage-mediated multi-copy drive systems to accumulate deactivating mutations.

On the other hand, the resolution of some intragenomic conflicts can transition to more complex genetic systems by acquiring new interacting components (figure 1*c*). In the murine clade of mice, there is an ongoing intragenomic conflict over transmission between sex chromosomes [36,38,39]. In this group, the Y chromosome of *Mus musculus* subspecies contains approximately 700 genes [52]. This is in striking contrast with most mammalian Y chromosomes, which are characterized by accumulation of repeats and loss of most of the ancestral genes present prior to the formation of sex chromosomes [41,45,107]. In the mouse Y chromosome, only 2% of the ancestral genes remain—the rest of the genes correspond to four secondarily acquired and massively amplified multigene families: *Sly*, *Srsy*, *Ssty1* and *Ssty2* [52]. Each of these genes has co-amplified paralogs on the X chromosome [108]. The most common organization of the almost 90 Mb of acquired sequence in the mouse Y chromosome is composed of a 500 kb-long tandemly repeated unit, each containing a copy of *Sly*, *Srsy* and *Ssty1/2* [52]. However, these genes were acquired at different time points during the evolutionary history of the group [109]. Therefore, the higher-order-repeat organization likely evolved afterwards and might help facilitate the maintenance of dosage and/or stoichiometry of gene products. In turn, the X-linked paralogs of these gene families (*Slx/Slx1*, *Srsx* and *Sstx*) are distributed in independent, rather than interleaved, clusters on the X chromosome [108,110]. These organizational differences may be driven by unique mutational patterns on the Y chromosome (see below).

### (b) Mutation bias and conflict fuel rapid Y chromosome evolution

Because of their high repeat content, the structural organization of Y chromosomes is evolutionarily labile and prone to rearrangements (e.g. [61]), even within species [111]. Structural variations might have functional consequences: variation in Y-linked heterochromatin contributes to variation in gene expression genome-wide (e.g. [112–114]) and resistance to meiotic drive [111,115]. Beyond the amplification of repeated sequences through unequal exchange, the Y chromosome has unusual mutation properties that can create more dramatic rearrangements. In most species with differentiated sex chromosomes, the Y chromosome has few regions of homology shared with the X chromosome, leaving limited options for homologous exchange between the chromosomes. The alternative to homology-directed repair is non-homologous end joining (NHEJ), which is more error-prone, often resulting in 1–2 bp indels. However, an important component of NHEJ machinery appears to be excluded from heterochromatin [116]. This condition may create an interesting situation for Y chromosomes, which are rich in heterochromatin and lack a homologue for homology-directed repair [61]. In the absence of core NHEJ components, Y chromosomes may use more error-prone mechanisms of double-strand break (DSB) repair, like microhomology-mediated end joining (MMEJ) [61]. MMEJ can cause large deletions and complex structural rearrangements, including translocations and telomere fusions [117]. Consistent with these predictions, Chang *et al*. [61] found unique mutation signatures (mutation spectra skewed toward larger deletions and indels with regions of microhomology) on the Y chromosomes of *D. melanogaster*, *D. simulans*, *D. sechellia* and *D. mauritiana*.

Other Y chromosome features may cause differences in the spectrum of mutations. For example, many Y chromosomes are rich in simple tandem repeats, which can lead to elevated numbers of DSBs. The excess of DSBs can increase the use of the MMEJ pathway [118,119], and thus elevated mutation. Despite the presumed consequences of having error-prone mechanisms of DNA repair, the alternative—homologous recombination between repeats in different genome regions—may have even more profound consequences for genome organization and stability. The lower gene density and specialization of Y-linked genes on male-specific functions may lead to relaxed constraints and make the Y chromosome permissive to high rates of structural evolution. Therefore, Y chromosomes have properties that could compensate for the smaller effective size and inefficacy of selection by exploring a wider mutational space. Structural evolution, like variation in copy number, involves mutations of large effect size and provides an opportunity to quickly respond to intense selective pressures (like the ones caused by drive). For example, the independent parallel evolution of insecticide resistance in diverse insects features gene duplications and structural mutations caused by transposable element insertions [120].

The tit-for-tat dynamics of these conflicts can lead to rapid demographic changes in populations and have negative consequences for the genetic diversity of sex chromosomes. Selective pressure against the pleiotropic effects of meiotic drive and Fisherian sex-ratio selection can maintain a balanced copy number between paralogs. Consistent with this idea, *Slx* and *Sly* copy number correlates across natural populations of mice [121,122]. However, crosses between populations or species at contact zones could produce individuals with unbalanced copy numbers. The introgressed high copy-number chromosomes will, therefore, quickly spread in the naive population [122,123]. Repeated bouts of rapid sequence turnover can cause selective sweeps; population genomic signatures indistinguishable from those produced by organismal adaptive evolution [124–126], and therefore require additional study to be associated with genetic conflicts [127].

## 4. A framework for conflict-mediated evolution of multigene families on sex chromosomes

What are the mechanisms driving the co-amplification of gene families on sex chromosomes? The evidence reviewed here suggests, as others pointed out [36,37,128], an intragenomic conflict over transmission in the heterogametic sex. However, direct evidence for meiotic drive (*sensu lato*; [4]) is limited to a few systems. In mice, copy number imbalance between the X- and Y-linked paralogs, *Slx-1* and *Sly*, produces distorted sex-ratios in the direction of the chromosome with higher copy number or expression level [38,39,129]. In *D. melanogaster*, *Ste* overexpression was also associated with drive [35,101]—deletions at the *Su(Ste)* locus severely impair male fertility, exacerbate non-disjunction and seem to cause higher X-bearing surviving sperm count [101].

One intriguing feature of sex-ratio drive is the difference in the nature of suppression or resistance across systems: while some systems evolved autosomal, in addition to Y-linked, suppressors or resistance (e.g. [111,115,130]), others show no autosomal suppression (see [22]), or suppression is completely absent (e.g. [22,131]). These contrasts in drive and suppression dynamics may suggest differences in the intrinsic properties of these drive systems (e.g. [131]). In this context, we propose a framework that can explain the evolution of X/Y co-amplification of gene families, given their genomic features. The model assumes amplification driven by countervailing selective pressure in a dosage-mediated arms race (i.e. dose-dependent amplification of drive and suppressor loci with additive effects). If the distortion caused by, say, the X chromosome is counteracted by a suppressing (or counter-driving) Y chromosome in a dosage-sensitive fashion, some conditions can allow repeated cycles of expansion (figure 1). Hurst & Pomiankowski [18] explored the different conditions for the spread of drivers and suppressors on autosomes versus the chromosome directly affected by drive. Consider a population that segregates for a pair of alleles—sensitive or insensitive—at a target locus, with frequencies *γ* and 1 − *γ*. As sex chromosomes do not normally undergo recombination, a new X-linked driver that targets all Y chromosomes can readily spread in the population, as long as it is linked to an insensitive allele [18]. The driver will invade the population when

$$s\;<\;1\;-\;\frac{1\;+\;r(1-\gamma )}{2k[1-r(1-\gamma )]},$$

[18,20,132]. Here, *s* is the fitness cost to male fertility, *r* is the recombination rate between the driver and the sensitivity loci and *k* is the strength of the drive (i.e. 0.5 = no distortion). The recombination rate determines how often ‘suicidal’ combinations are created [10], causing the X to drive against itself. This scenario applies for species with undifferentiated or early neo-sex chromosomes—homology between sex chromosomes increases the likelihood of both sharing the target loci [42]. In species with ‘old’ sex chromosomes, however, it is reasonable to assume that there is no X-linked target locus, which simplifies the equation [20]:

$$s\;<\;1\;-\;\frac{1}{2k}.$$

Now let us consider the evolution of modifiers that neutralize the distortion. Sex-linked meiotic drive elements have negative fitness effects. The basis of distortion in species with heterogametic males is associated with abnormalities or disturbances in meiosis and/or gametogenesis; therefore, it imposes a cost to male fertility [20,23]. In addition, linked deleterious mutations can hitchhike with the driver (see [25]). Finally, drive also produces skewed sex-ratios in the offspring; this could cause populations to go extinct [15,21]. This effect is even more dramatic with Y-linked drivers given their uniparental inheritance. Driving Y chromosomes, in turn, do so in about one-third of the time of a driving X chromosome [15]. Sex-ratios are under frequency-dependent selection—when sex-ratios are increasingly distorted, the relative reproductive value of the most common sex decreases [26]. This generates selective pressure for the spread of alleles that produce more of the rarer sex, restoring the approximately 1:1 optimal Fisherian sex-ratio [15,30]. For these reasons, as a sex-linked driver spreads, it generates selective pressure for the evolution of unlinked suppressors on autosomes and the affected sex chromosome [6,133]. As Hurst & Pomiankowski [18] showed, however, the conditions for spread of one versus the other differ. Selection for sex-linked suppressors is stronger than autosomal ones when the driver is rare. This result is intuitive—when the driver is at low frequency in the population, sex-ratio is not greatly distorted and autosomes spend roughly half of the time in individuals of the opposite sex. The sex chromosome being targeted by the driver, in turn, suffers the negative pleiotropic effects of drive and is excluded from gametes *every* time that is paired with the driver. As the driver sweeps throughout the population, the sex-ratio is increasingly distorted and Fisherian sex-ratio selection makes autosomal suppressors equally favoured [18].

In this scenario, the evolution of co-amplified gene families on sex chromosomes may be explained by a model that assumes a dosage-mediated conflict over transmission. In this model, a mutation that causes drive arises on a sex chromosome (most likely the X; see [20]). The origin could be due to a (retro)duplication of an autosomal gene (e.g. *Ssl* [61]) or the formation of neo-sex chromosomes by the fusion between an autosome and one sex chromosome (e.g. [55,65]). The mutational target size is higher in recently formed neo-sex chromosomes, as the immediate sex-linkage of an entire autosomal gene set provides abundant substrate for both mutation to drivers and suppressors, reducing the waiting time for them to arrive by other mechanisms like a gene duplication from a different chromosome.

**Figure 2.**
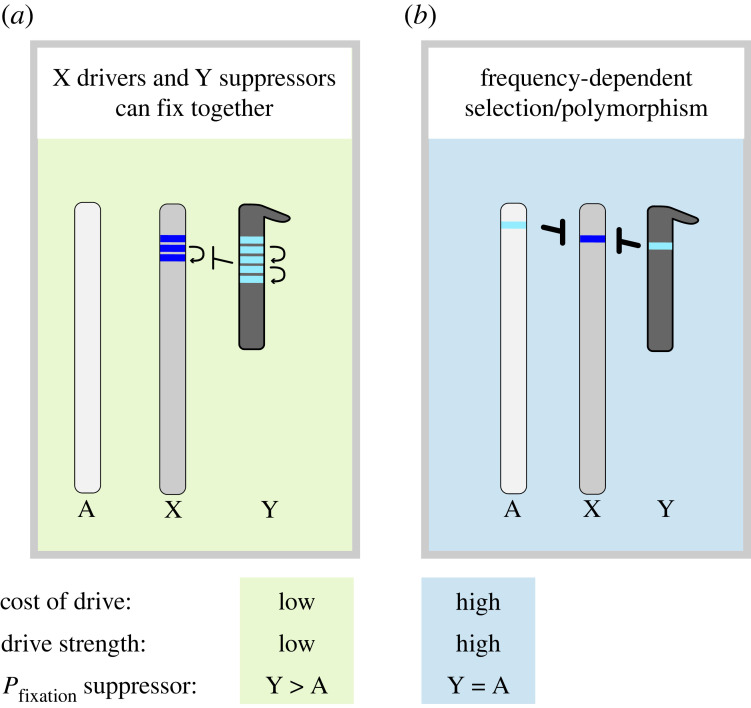
Different conditions for the emergence and spread of drive and suppression on Y chromosomes relative to autosomes. (*a*) A Y-linked suppressor enjoys a shorter transit time than an autosomal one when the cost and strength (*k*) of the driver are low. Autosomes spend half of their time in females and males, and therefore an autosomal suppressor is ‘seen’ by selection only half as frequently as a Y-linked one. When the cost and strength of drive are low, an X-linked driver and Y-linked suppressor can spread to fixation together. These conditions might allow the emergence of paralogous ampliconic gene families by recurrent bouts of fixation of duplicated drivers and suppressors. (*b*) Alternatively, consider a population that quickly approaches distorted sex-ratios due to a strong driver. In this case, the strong selective pressure to re-establish optimal sex-ratios and against the deleterious pleiotropic effects of the driver make the recruitment and spread of Y-linked and autosomal suppressors similarly likely. Under this scenario, neither the driver nor suppressor can spread to fixation. Since sex-ratio is a trait under frequency-dependent selection, they remain polymorphic.

Let's assume that the distortion caused by the driver is weak enough—and therefore, population sex-ratios are not strongly skewed—but individuals carrying the driver still suffer deleterious pleiotropic effects. The selective pressure for acquiring suppressors is going to be stronger in the chromosome that is being excluded from gametes than in any autosome (see above; [18]). Given enough evolutionary time, a mutation that makes a Y chromosome insensitive, resistant, or counteracting to the effects of the driver will spread through the population (figure 2; [18]). Consistent with theoretical expectations, Vaz & Carvalho [134] showed that an X-linked driver and a Y-linked suppressor can spread to fixation together as long as the former is not too deleterious and the latter is neutral. By contrast, the conditions for the fixation of autosomal suppressors are more restrictive (figure 2; [29]). Together, these conditions can promote recurrent bouts of fixation of X-linked drivers and Y-linked suppressors [135]. If rearrangements such as duplications are not limiting, this process can drive the expansion of genes caught in the conflict, in a *stepwise* fashion, leading to the formation of ampliconic gene families.

## 5. Conclusion

In the past 20 years, advances in genome sequencing helped reveal a recurring pattern on sex chromosomes: X and Y chromosomes independently acquire and co-amplify gene families. Some of the features of these co-amplified gene families are difficult to explain in light of ecological adaptation—they are paralogous, lineage-specific with rapid turnover, have testis-specific expression, some produce small RNAs, and in some species are associated with distorted sex-ratios. Instead, these features are consistent with a history of intragenomic conflicts over transmission through the male germline [37]. The dynamics of these conflicts can be exceptionally fast and have profound consequences for genome evolution. The unique mutational properties of the Y chromosome may exacerbate this effect, facilitating the rapid increase in copy number, or providing structural changes that trigger the production of small RNAs. Given their properties, the origin of ampliconic structures may be explained by the recurrent fixation of mutations of small, additive effects on drive and suppression (or counteracting drive) between sex chromosomes.

To our knowledge, no genomic analyses to date reveal evidence for co-amplified sex-linked gene families in species with ZW sex determination systems (i.e. ZZ males and ZW females). This may simply be a result of ascertainment bias—ZW systems have received less attention than their XY counterparts, probably due to the dearth of model organisms with ZW determination [136]. On the other hand, this may also be due to differences between male and female meiosis in selection pressures and opportunities for conflict. Female meiosis has an asymmetry that meiotic drivers can exploit—only one allele gets recruited to the functional pole—therefore, selfish centromeres that control chromosome segregation can hijack meiosis to bias their own segregation to the developing oocyte. Here the cheaters tend to involve repetitive DNAs at centromeres rather than protein coding sequence (e.g. [137]). However, one well-studied autosomal female meiotic drive system in maize, abnormal chromosome 10 (Ab10) [138], involves ‘knobs’ of repetitive DNA that can drive in female meiosis through neocentromere activity. Although this system is not sex-linked, it presents remarkable parallels with some features of XY co-ampliconic genes. First, a cluster of ampliconic genes, *Kinesin driver* (*Kindr*), is linked to the driver and is necessary for neocentromere motility and segregation distortion; and second, an ampliconic locus, paralogous to *Kindr*, exists in repulsion LD to the drive. These paralogs consist of non-coding ‘pseudo-*Kindr*’ repeats that produce small interfering RNAs that are candidate suppressors of *Kindr* [138,139]. If they exist, ZW co-ampliconic genes driven by intragenomic conflicts over transmission may have similar dynamics to Ab10 and the systems reviewed here.

Meiotic drive is an evolutionary force with far-reaching evolutionary consequences [4]—but how widespread is it in nature? In the almost 100 years since its first report [21], we have seen many cases of meiotic drive and made significant progress in understanding the molecular basis of distortion. However, most reported cases are of strong drive and limited to a handful of taxa. For instance, a driver with a transmission advantage of less than 2% could have profound evolutionary consequences, but it would be difficult to detect in the laboratory as this requires careful genetic experimentation (e.g. [67,140]). Considering these experimental limitations, testing the pervasiveness of intragenomic conflicts thus rests, for now, on more circumstantial evidence. ‘Genomic signatures’ of conflict can be complementary to other approaches, like selective sweeps scans, which on their own are confounded with classic modes of adaptation. The emerging view is that meiotic drive is not the oddity it was once considered—it may be more pervasive than previously appreciated. Improving genomic methods and genetic approaches will provide insights on the extent and influence of conflicts on genome evolution. Sequencing technologies and genome assembly approaches have seen a great deal of improvement in the past decade. The broader accessibility of these resources will allow us to explore these ‘signatures’ (e.g. ampliconic sequences, X/Y co-amplification of paralogs, rapid evolution, production of small RNA) in currently underrepresented taxa. Advances in genome editing and functional genomic approaches should also allow for unprecedented insights into the genomic architecture and molecular mechanisms of drive in model and non-model organisms. For example, CRISPR-mediated deletion of multi-copy gene families allows for the genetic and functional dissection of sex-linked drive components in mice and should be feasible in non-model organisms. These technologies may usher in the next phase of genetic scrutiny of drive systems from a wider range of study systems.

## Data Availability

This article has no additional data.
